# Case Report: Longitudinal Extensive Transverse Myelitis With Novel Autoantibodies Following Two Rounds of Pembrolizumab

**DOI:** 10.3389/fneur.2021.655283

**Published:** 2021-04-30

**Authors:** Salma Charabi, Lotte Engell-Noerregaard, Anna Christine Nilsson, Christian Stenör

**Affiliations:** ^1^Department of Neurology, University of Copenhagen Herlev Hospital, Herlev, Denmark; ^2^Department of Oncology, University of Copenhagen Herlev Hospital, Herlev, Denmark; ^3^Autoimmune Laboratory, Department of Clinical Immunology, Odense University Hospital, Odense, Denmark; ^4^Faculty of Health and Medical Sciences, University of Copenhagen, Copenhagen, Denmark

**Keywords:** immune checkpoint inhibitors, transverse myelitis, immune related adverse events, pembrolizumab, case report (source: MeSH NLM), neurological immune-related adverse effects, PD-1 monoclonal antibody, longitudinal extensive transverse myelitis

## Abstract

A 63-year-old male with metastatic non-small cell lung cancer developed longitudinal extensive transverse myelitis (LETM) following two cycles of Pembrolizumab, an immune checkpoint inhibitor (ICI) targeting the programmed cell death receptor 1 (PD-1). Magnetic resonance imaging (MRI) showed centromedullary contrast enhancement at several levels, cerebrospinal fluid (CSF) cytology showed lymphocytic pleocytosis, and indirect immunofluorescence assay (IFA) on the primate cerebellum, pancreas, and intestine revealed strong binding of neuronal autoantibodies to unknown antigens. CSF C–X–C motif ligand 13 (CXCL13) was elevated. The patient was treated with plasma exchange (PEX) and intravenous (i.v.) methylprednisolone (MP) 1 g/day for 5 days followed by oral (p.o.) MP 100 mg/day for 10 days with clinical and radiological response. However, after discontinuation of MP, LETM relapsed and the patient developed paralytic ileus presumably due to autoimmune enteropathy and suffered a fatal gastrointestinal sepsis. Findings of novel neuronal autoantibodies and highly elevated CXCL13 in CSF suggest that the severe neurological immune-related adverse event (nirAE) was B-cell mediated contrary to the commonly assumed ICI-induced T-cell toxicity. An individual evaluation of the underlying pathophysiology behind rare nirAEs is essential for choosing treatment regimens and securing optimal outcome.

## Introduction

Immune checkpoint inhibitors (ICIs) have changed the way we approach cancer treatment. Instead of attacking the tumor tissue itself, ICIs enhance the endogenous antitumor response by blocking inhibitory antigens CTLA-4 (cytotoxic T-lymphocyte-associated protein) or PD-1 (programmed cell death protein 1) expressed on host immune cells or programmed cell death protein ligand (PD-L1) on some tumor cells. Pembrolizumab is an anti PD-1 antibody that has shown remarkable results in the treatment of many solid cancers including malignant melanoma and non-small cell lung cancer (NSCLC), improving the overall survival (OS) and progression-free survival (PFS) compared to conventional therapies (1, 2).

While treatment effects can be significant, ICIs can induce immune-related toxicity in almost all tissues, most commonly in the gastrointestinal tract, skin, liver, endocrine, and pulmonary organs (3). The neurological immune-related adverse events (nirAEs) are less common with an overall incidence between 3.8 and 12% from randomized trials—highest with combination therapy (4). Serious nirAEs defined as Common Terminology Criteria for Adverse Events (CTCAE) grades 3–5 are below 1%, and to our knowledge, only two case reports have been published with ICI-induced transverse myelitis (5, 6). However, many cases of myelitis are reported as encephalitis or encephalomyelitis in clinical trials, which makes accurate incidence difficult to estimate (7). A recent review of data from World Health Organization's (WHO's) pharmacovigilance database, VigiBase, found 24 cases reported as ICI-related myelitis including four cases with encephalomyelitis (8). To our knowledge, this is the third case report describing transverse myelitis following monotherapy with ICIs and the second case with evidence of autoreactive antibodies targeting unknown neural antigens.

## Case Description

A 63-year-old male was diagnosed with metastatic pulmonary squamous cell carcinoma, T4N3M1c, PD-L1 expression >50%. He suffered from chronic obstructive pulmonary disorder (COPD), was a former smoker with 33 pack years, and had performance status 1. Comorbidities were hypertension and hypercholesterolemia, but he had no known underlying autoimmune diseases or family history of such. Earlier the same year, he had undergone surgical excision of a malignant melanoma (T1aN0M0) with no adjuvant treatment. His pulmonary cancer was treated with two cycles of first-line treatment with Pembrolizumab (a PD-1 inhibitor) 2 mg/kg with a 3-week interval.

One day after his second treatment with Pembrolizumab, he presented with fever (38.9°C), chills, intermittent headache, and pain located to joints and lower back. During the following week, he developed constipation and urinary retention to which he was catheterized several times in the ER. After 2 weeks of urinary retention and obstipation, the patient was admitted due to progressively painful dysphagia with hoarseness and burping tendency. A gastroscopy showed no signs of pathology, symptoms relieved with fluconazole treatment, and he was discharged.

**Figure 1:**
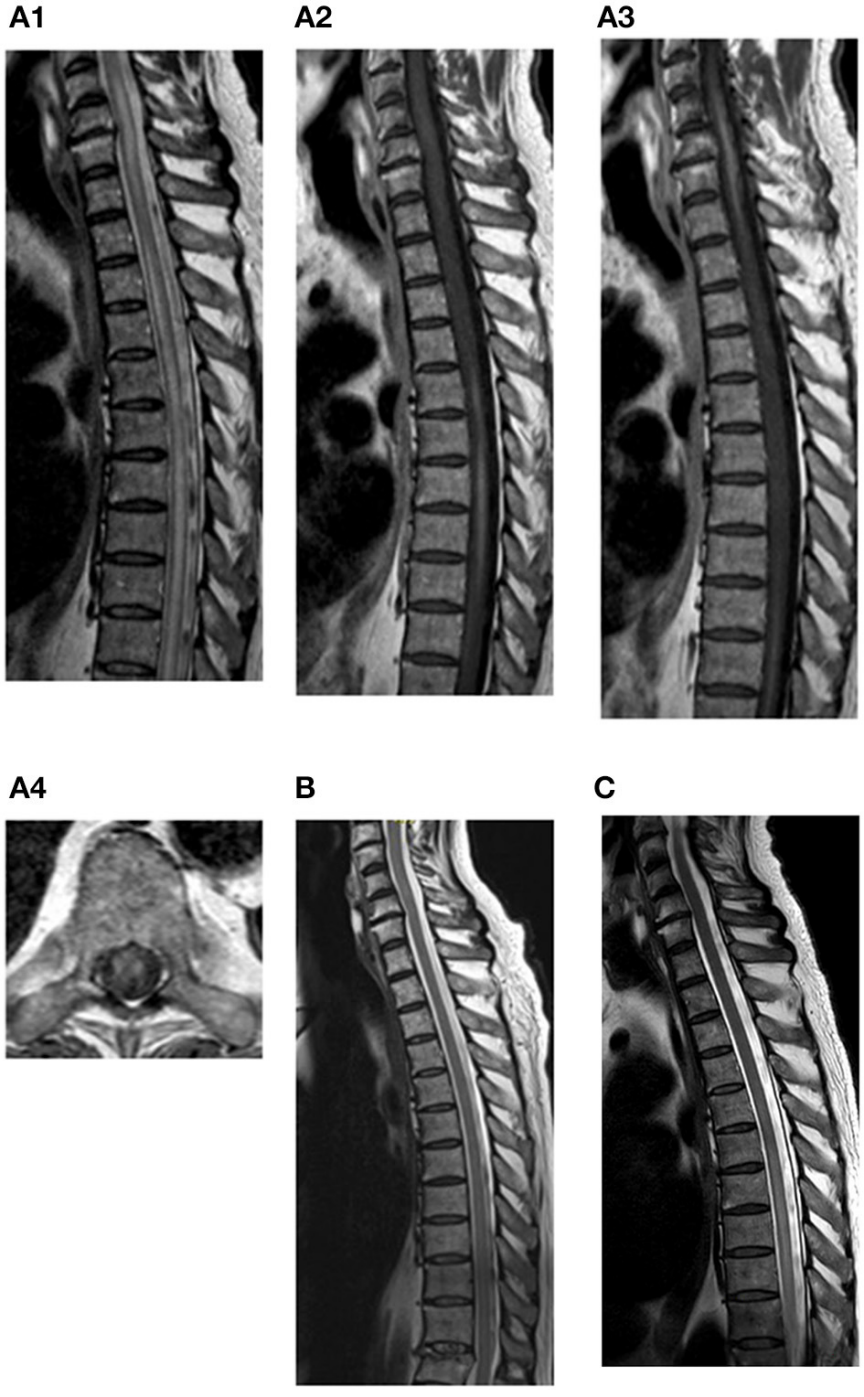
MRI progression: at time of diagnosis **(A1–4)**, 3 days after last dose of p.o. MP **(B)** and 11 days after last dose of p.o. MP **(C)**. **(A1)** Sagittal T2-weighted MRI of columna totalis showing extensive LETM at cervical and thoracic levels. **(A2)** Sagittal T1-weighted MRI of columna totalis with contrast showing contrast enhancement at levels Th5–Th9. **(A3)** Sagittal T1-weighted MRI with contrast showing enhancement from C4-Th1. **(A4)** Axial T1-weighted MRI with contrast showing anterolateral multifocal contrast enhancement at level Th9. **(B)** Sagittal T2-weighted MRI of columna totalis showing almost complete regression of LETM. No contrast enhancement on T1 (not shown). **(C)** Sagittal T2-weighted MRI of columna totalis with relapsing LETM on levels Th5–Th11. Contrast enhancement on T1 weighted MRI (not shown).

Five days later, the patient was readmitted due to lumbar fatigue, reduced muscle strength in the lower extremities, and difficulties walking. A neurological examination showed a flaccid tetraparesis with greater affection of the lower extremities, most prominently bilaterally in the hip and ankle joints. Deep tendon reflexes were normal, and sensibility was not affected. An MRI without contrast showed suspicion of LETM, and p.o. Prednisone 50 mg/day was initiated. Also, a CT scan of the lungs showed significant regression in the pulmonary cancer compared to baseline PET-CT. A few days after, the patient could not walk and was immobilized to a wheelchair or bed rest. While an MRI of the cerebrum showed no signs of pathology, a repeat MRI of the spinal column with contrast confirmed LETM (Figure 1A). Subsequently, treatment with bolus i.v. MP 80 mg followed by i.v. MP 1 g/day for 5 days and PEX every second day for 2 weeks was initiated.

On the second day of the initiated treatment (after two doses of i.v. MP 1 g and 12 h after first PEX), blood and CSF were drawn for analysis. Blood was tested for autoantibodies related to LETM, but both anti-aquaporin-4 (AQP4) and anti-myelin oligodendrocyte (MOG) antibodies were negative. CSF revealed that lymphocytic pleocytosis (63 cells with 50 lymphocytes) significantly elevated IgG, IgG index, and elevated CXCL13 in CSF (Table 1). CSF cultures and polymerase chain reaction (PCR) showed no signs of bacterial or fungal growth or viral activity. CSF flow cytometry showed an overall normal distribution of T- and B-cells; however, there was a population of CD38^+^ cells, presumably plasma cells.

**Table 1:** CSF and serum findings after two doses of 1 g i.v. MP and one treatment with PEX.

**CSF**	**Result**	**(Reference value)**	**Serum**	**Result**	**(Reference value)**
Albumin	420 mg/L	(100–370)	AQP-4-Ab	Neg	(<1:10)
Glucose	5.0 mmol/L	(2.2–3.9)	MOG-Ab	Neg	(<1:10)
Protein	0.80 g/L	(0.15–0.50)	NACHRA3-Ab (IgG)	Neg	(<0.05 nmol/L)
IgG	248 mg/L	(14–52)	Calcium channel P/Q-type	Neg	(<40 pmol/L)
Cells	63 e6/L	(<5)	DPPX	Neg	(Neg)
– Lymphocytes	50 e6/L	(<5)			
– Macrophages	3 e6/L	None	*Paraneoplastic panel*		
– Neutrophils	<1 e6/L	(<1)	IFA on primate cerebellum	Pos, 1:100	(<1:10)
CXCL13	119 ng/L	(<20)	IFA on primate intestine	Pos, 1:100	(<1:10)
Oligoclonal bands	Neg	(Neg)	IFA on primate pancreas	Pos, 1:100	(<1:10)
DPPX	Neg	(Neg)	EUROLINE PNS 12 Ag® LIA	Neg	(Neg)
			GAD65 Ab	Neg	(<1:10)
*Paraneoplastic panel*					
IFA on primate cerebellum	Pos	(Neg)			
IFA on primate intestine	Pos	(Neg)			
IFA on primate pancreas	Pos	(Neg)			
EUROLINE PNS 12 Ag® LIA	Neg	(Neg)			
GAD65 Ab	Neg	(Neg)			
IgG index	1.64	(0.38–0.67)			

*IgG, immunoglobulin G; CXCL-13, C–X–C motif chemokine ligand 13; AQP-4-IgG, Aquaporine 4 IgG; MOG-Ab, myelin oligodendrocyte glycoprotein antibodies; NACHRA3-AG, neuronal acetylcholine receptor subunit alpha-3-antibody; Anti-GFAP, anti-glial fibrillary acidic protein antibody; IFA, immunofluorescence assay; PNS 12 Ag® LIA, paraneoplastic Neurological Antigens 12 Profile line immunoassay (testing for amphiphysin, CV2, Ma/Ta, Ri, Yo, Hu, recoverin, Sox1, titin, Zic4, GAD and Tr antibodies); GAD65 Ab, glutamic acid decarboxylase-65 antibody*.

Indirect immunofluorescence assay (IFA) on primate cerebellar sections (Euroimmun AG, Luebeck, Germany) showed cytoplasmic and dendritic fluorescence of Purkinje cells and a granular fluorescence of the molecular and granular cerebellar layers in both serum and CSF. Euroline Paraneoplastic Neurological Antigens 12 Profile line immunoassay (LIA) (Euroimmun AG, Luebeck, Germany) was negative (testing for amphiphysin, CV2, Ma/Ta, Ri, Yo, Hu, recoverin, Sox1, titin, Zic4, GAD, and Tr antibodies). There was no indication of anti-glial fibrillary acidic protein (GFAP) antibodies on the tissue-based assay. Samples were sent to a reference laboratory for second opinion. An anti-neuronal reaction was confirmed; however, target antigen and hence clinical relevance could not be determined. Furthermore, there was a strong fluorescence of pancreatic islet cells and the cytoplasm of neurons in the intestinal plexus myentericus. Again, target antigens could not be identified, especially anti-GAD65 by enzyme-linked immunosorbent assay (ELISA) (Euroimmun AG, Luebeck, Germany) which was negative.

**Figure 2:**
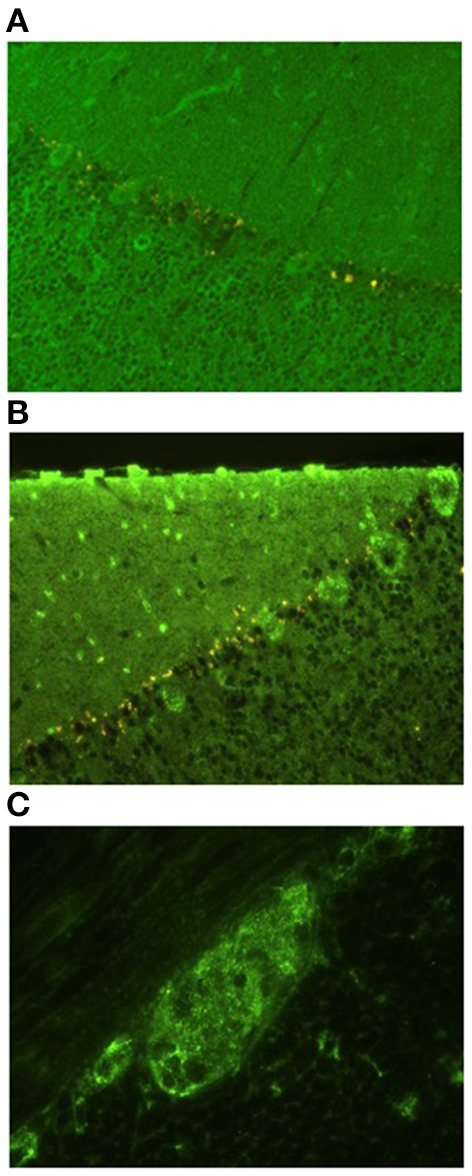
Indirect immunofluorescence assay on primate cerebellar and intestinal sections. **(A,B)** Granular cytoplasmic and dendritic fluorescence of Purkinje cells and a granular fluorescence of the molecular and granular cerebellar layers. **(A)** Sera in dilution 1:100, **(B)** undiluted CSF. **(C)** Granular cytoplasmic fluorescens of neurons in the intestinal plexus myentericus. Undiluted CSF.

After 5 days of i.v. MP 1 g/day, PEX was discontinued (after two series) due to markedly improved motor function and ability to walk again with a high walking frame. Corticosteroids were changed to p.o. MP 100 mg/day for 10 days without a taper. LETM remitted during treatment and MRI confirmed LETM regression (Figure 2B). The patient was subsequently discharged. However, at discharge the patient still suffered from constipation and urinary retention.

**Figure 3:**
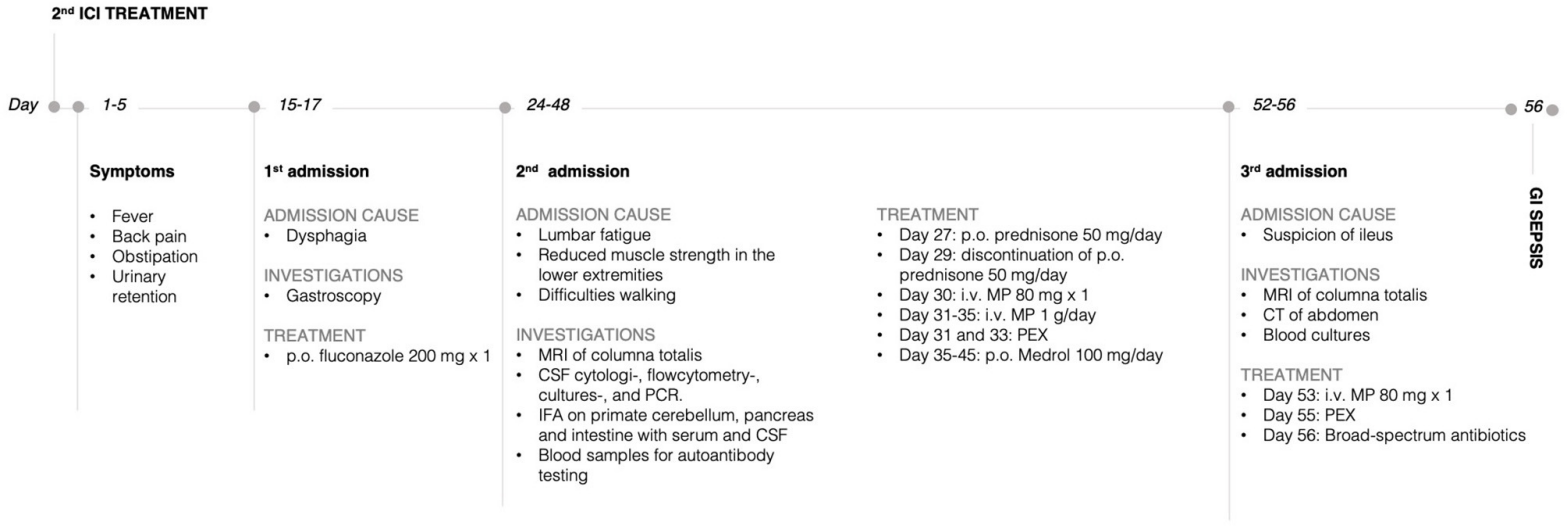
Timeline with selected events and data. p.o., oral; i.v., intravenous; MRI, magnetic resonance imaging; CSF, cerebrospinal fluid; IFA, indirect immunofluorescence assay; MP, methylprednisolone; PEX, plasmapheresis; CT, computer tomography; GI, gastrointestinal.

A few days later, the patient was readmitted with severe constipation. CT showed signs of paralytic ileus and an MRI showed relapsing LETM (Figure 2C). Treatment with i.v. MP 1 g/day was reinitiated. Four days later, the patient became septic with respiratory insufficiency, hypotension, tachycardia, kidney failure, and blood cultures showing growth of Bacteroides fragilis. Broad-spectrum antibiotics was initiated, and PEX was attempted on an empiric basis. The following day, the patient died of respiratory insufficiency due to sepsis following paralytic ileus. Due to his severe paralytic ileus, a post-mortem supplementary search for autoimmune autonomic/enteric neuropathy was done, but serum neuronal acetylcholine receptor subunit alpha-3-antibody (NACHRA3-Ab) was negative. Also, dipeptidyl-peptidase-like protein 6 (DPPX) antibodies were negative. An overview of the timeline is illustrated in Figure 3.

## Discussion

Treatment with ICIs is rapidly emerging within the oncological field. Being fairly novel, not all uncommon and severe adverse events are known or thoroughly described. LETM is a very rare nirAE that can easily be overlooked in the initial phase.

There are several lines of notice that endorse that both LETM and paralytic ileus were induced by Pembrolizumab; it evolved in close proximity to ICI treatment, and the patient did not receive any other new medications or therapies. No infectious cause was found, and known markers of LETM, i.e., anti-MOG, anti-AQP4, and anti-GFAP (the last only tested by indirect immunofluorescence in a tissue-based assay, not cell-based assay), were negative (Table 1). There was evidence of an antibody-mediated anti-neuronal reaction by IFA on the primate cerebellum and intestine, significant intrathecal IgG synthesis (IgG index of 1.64), and significantly elevated CXCL13, altogether suggesting B-cell/antibody-mediated disease.

This case shares some key features with prior similar cases; Wilson et al. equally presented the finding of novel neural autoantibodies in a case of Pembrolizumab-induced LETM, which had a 4-week lag from treatment to symptom onset and responded to MP and PEX (6). A case of Pembrolizumab-induced neuromyelitis optica spectrum disorder (NMOSD) also remitted with MP and PEX (9), and a case of steroid-refractory Nivolumab-induced NMOSD improved on PEX as monotherapy (10). While a case of Ipilimumab-induced meningoencephalomyelitis did not attempt PEX treatment, they achieved remission with Infliximab and long-term Prednisone following non-response to IVIG and MP combination therapy (11).

In our case, circulating antibodies are thought to be accountable for the nirAE on two levels: the ICIs themselves (IgG antibodies) and their induction of an antibody-mediated toxicity. Therefore, depletion of antibodies by PEX should theoretically be the first-line treatment in similar cases of severe ICI-induced LETM. Our case supports this thesis, as the patient partially regained motor function after two treatments with PEX, although i.v. MP probably also contributed. While no randomized trials have confirmed the efficacy, it is increasingly apparent from other neurological antibody-mediated autoimmune diseases, e.g., NMOSD, that early PEX is an important factor in achieving a good outcome (9, 10).

Taking the American Society of Clinical Oncology's (ASCO's) guidelines for grade 3–4 irAEs into consideration, the right treatment was initiated upon diagnosis. However, it is recommended to taper corticosteroids over at least 4–6 weeks (12). Along with early discontinuation of PEX, this might explain why the LETM relapsed. ICIs are IgG antibodies with long half-lives, why tapering of corticosteroids and a complete PEX series are essential to avoid a delayed inflammatory flare. This underlines that high-grade nirAEs (grades 3–4) should not be regarded and treated as their more common idiopathic autoimmune or post-infectious disease counterparts.

This case had a fatal outcome despite the initiated combination treatment. After partial remission of LETM, the patient developed paralytic ileus, presumably due to enteric neuropathy as an additional nirAE to Pembrolizumab. No specific anti-enteric neuronal antibodies could be detected; however, IFA on the primate intestine showed granular cytoplasmic fluorescence of cells in the myenteric plexus (Figure 1). Although the target antigen and hence the clinical relevance of this finding is unknown, it seems unlikely that this finding is merely coincidental.

ICIs are known to enhance T-cell responses; thus, irAEs are expected to be T-cell mediated. However, this case along with prior cases (6, 9, 10) suggests an ICI-induced activation of antibody-mediated toxicity. While we and Wilson et al. described a 4-week lag from treatment initiation to symptom onset, a review from 2019 described a mean lag of 94.8 days in all central nervous system nirAE case reports (13). This discrepancy suggests that the underlying mechanisms differ. A 4-week lag from ICI administration to symptom onset probably does not allow significant levels of novel neuronal IgG antibodies to be formed, suggesting that latent humoral autoimmunity was demasked by Pembrolizumab (6). Wilson et al. found anti-human-IgG antibodies bound to a specific T-regulatory cell subpopulation in peripheral blood, suggesting a potential adverse target for Pembrolizumab and a possible link between ICIs and B-cell activation. Also, a study investigating early B-cell changes in combination ICI therapy found that a B-cell subset with properties of rapid activation specifically increased and correlated with high-grade irAE, whereas changes in T-, NK-, and myeloid cells did not (14). This suggests that B-cells at least in some cases can be held accountable for autoimmunity following ICI therapy.

Therefore, we speculate that it could be feasible to use B-cell-depleting treatments, e.g., Rituximab under continuation of ICI to allow continuous T-cell-mediated antitumor activity. Indeed, an experimental study with melanoma cancer cells showed that B-cell depletion had no effect on tumor growth, response to PD-1 inhibition, or survival rates (15).

In conclusion, as ICI emerges within the field of cancer treatment,this case emphasizes the need of a dedicated and specialized team to handle the wide range of rare irAEs. Anti-neuronal antibodies with an unknown target antigen were seen, suggesting an unknown underlying pathophysiology behind this nirAE. Further studies should investigate the role of B-cells in nirAEs and confirm the optimal treatment taking the effector mechanism into consideration.

## Data Availability Statement

The raw data supporting the conclusions of this article will be made available by the authors, without undue reservation.

## Ethics Statement

Ethical review and approval was not required for the study on human participants in accordance with the local legislation and institutional requirements. The patients/participants provided their written informed consent to participate in this study. Written informed consent was obtained from the individual(s) for the publication of any potentially identifiable images or data included in this article.

## Author Contributions

SC wrote the first draft and was the main editor of following revisions and revised all patient files and selected relevant events and data. Also, SC made Figure 3. CS discovered the case in clinics, made the diagnostics, and wrote the majority of sections with neurological content including Figure 1. AN performed the immunological testing and wrote the majority of sections with immunological content including Figure 2. LE-N was the primary oncologist in the terminal stage of the patient case and wrote sections of the manuscript. All authors contributed to manuscript revision, read, and approved the submitted version.

## Conflict of Interest

The authors declare that the research was conducted in the absence of any commercial or financial relationships that could be construed as a potential conflict of interest.
